# Misleading Claims About Tobacco Products in YouTube Videos: Experimental Effects of Misinformation on Unhealthy Attitudes

**DOI:** 10.2196/jmir.9959

**Published:** 2018-06-29

**Authors:** Dolores Albarracin, Daniel Romer, Christopher Jones, Kathleen Hall Jamieson, Patrick Jamieson

**Affiliations:** ^1^ University of Illinois at Urbana Champaign Champaign, IL United States; ^2^ Annenberg Public Policy Center University of Pennsylvania Philadelphia, PA United States

**Keywords:** health communication, tobacco

## Abstract

**Background:**

Recent content analyses of YouTube postings reveal a proliferation of user generated videos with misleading statements about the health consequences of various types of nontraditional tobacco use (eg, electronic cigarettes; e-cigarettes).

**Objective:**

This research was aimed at obtaining evidence about the potential effects of YouTube postings about tobacco products on viewers' attitudes toward these products.

**Methods:**

A sample of young adults recruited online (N=350) viewed one of four highly viewed YouTube videos containing misleading health statements about chewing tobacco, e-cigarettes, hookahs, and pipe smoking, as well as a control YouTube video unrelated to tobacco products.

**Results:**

The videos about e-cigarettes and hookahs led to more positive attitudes toward the featured products than did control videos. However, these effects did not fully translate into attitudes toward combustive cigarette smoking, although the pipe video led to more positive attitudes toward combustive smoking than did the chewing and the hookah videos, and the e-cigarette video led to more positive attitudes toward combustive cigarette smoking than did the chewing video.

**Conclusions:**

This research revealed young people’s reactions to misleading claims about tobacco products featured in popular YouTube videos. Policy implications are discussed.

## Introduction

The study of media influences on smoking among adolescents and young adults has a long history of uncovering significant health threats [1,2]. Although these findings, along with regulatory efforts, have contributed to the decline of tobacco portrayal (mostly cigarettes) in cinema and on television since 1950 [3,4], emerging media such as the internet remain largely unregulated [5,6]. For example, many noncommercial internet materials generated by community members minimize or misrepresent the negative health consequences of tobacco use, either through omission (eg, not noting the negative health consequences [5,7-9]), or through commission (eg, asserting that smoking is safe or even has health benefits [9-11]). The question guiding this research is:

What is the likely effect of such tobacco- friendly communications disseminated informally on the internet?

There is a rising suspicion that online exposure to user-generated content on YouTube shapes young people’s perceptions of tobacco [5-15]. There are at least three reasons for this concern. First, there are large numbers of tobacco-related messages on YouTube, with more messages presenting favorable rather than unfavorable views on tobacco [6-10,12,13,15-17]. Second, YouTube reports over one billion users who collectively watch hundreds of millions of hours of video per day [18]. Third, according to a recent survey, half of today’s teens cite YouTube as their favorite website [19]. Despite the potential influence of this large number of regularly viewed messages from an outlet that young people trust, little systematic research has elucidated the degree to which YouTube messages influence attitudes toward tobacco products. Such evidence is critical for future policy decisions about tobacco-related content presented on the internet [11,12,20].

Prior studies of tobacco messages on YouTube have provided invaluable qualitative analyses of content [6,7,10,12,13,16] and determined prevalence of tobacco messages [21]. The next step, however, is to ascertain if these messages can promote favorable attitudes toward tobacco products such as electronic cigarettes (e-cigarettes) and combustive cigarette smoking in young viewers. Research on alcohol use portrayals in social media has already shown harmful influences of internet content [22-24]. In the arena of tobacco, the most likely targets of influence are products of ambiguous health consequences in the eyes of the public. Recent surveys suggest that young adults regularly use one or more tobacco products such as hookahs and e-cigarettes [25-27], even though combustive cigarette use has declined [3,27]. Thus, we identified popular user-generated YouTube videos that contained misleading messages about products such as chewing tobacco, hookahs, and e-cigarettes. We then experimentally examined whether these videos create favorable attitudes toward the featured product. We focused on four different tobacco products: (1) chewing tobacco, (2) e-cigarettes, (3) hookahs, and (4) pipe smoking. Four highly viewed messages were selected and presented online to a sample of 18-to-24-year olds (N=350) with varied prior use of tobacco products.

## Methods

### Sample and Experimental Design

Four hundred and thirty participants aged 18-24 years in the United States completed the 15-minute study via Amazon Mechanical Turk [28]. The study was approved by the Institutional Review Board of the University of Pennsylvania. Participants were compensated US $1 for study completion. Researchers have found psychometric indicators of quality of Mechanical Turk data to be comparable to subject pools at research universities [29]. Nevertheless, we included two checks on participation quality [30]. To ensure that participants read the instructions, they were required to answer a question about their favorite color by clicking green and pink regardless of their actual preferences. Failing to follow this instruction indicates that this, and possibly other instructions of questions, were not read. A second check ensured that participants actually watched the videos by indicating what was discussed. Participants responded to a checklist of products including the tobacco product that appeared in the video and indicated which products appeared or were mentioned. Participants failing either check (80/430, 18.6%) were excluded, producing a final sample size of 350. Comparisons between the excluded and retained participants indicated no significant differences in tobacco consumption or demographics.

### Selection of Videos

A search of popular videos on YouTube using 136 tobacco-related search terms identified over 8000 videos after removing unrelated content. Search terms included **“**smoke,” “smoking,” “tobacco,” “cigarillo,” and colloquial terms for products such as “shisha” for hookah tobacco. Criteria for exclusion included the video having fewer than 20,000 views at the time of download, the video being in a language other than English or having no audio, a video not containing tobacco content, or a video not being retrievable due to a broken or inactive ink. Using an Excel random number generator, of the 8000 eligible videos, 200 were selected for further coding. Three coders met a Krippendorff alpha reliability of K alpha >.91 for the classifications of videos into different types of claims. This coding identified four major types of misleading health messages from this sample of YouTube videos: (1) rejection of science (ie, evidence supporting the harmfulness of a tobacco product is faulty), (2) assertion of benefit (eg, tobacco can be healthy), (3) denial of harm (eg, tobacco is not harmful after all), or (4) presence of acceptable risks (eg, using tobacco is no riskier than other common activities).

We pinpointed 37 videos containing misleading portrayals of tobacco’s health consequences that lacked discernable brand affiliation or sponsorship. To ensure a varied sample of contents, we selected four videos representing each of the misleading categories. Within this selected set, two of the videos featured young adult white males and two featured adult white males. Within the selected set, videos were also representative of the major categories of misleading health claims that reached large audiences. In the selected videos, the source: (1) claimed that drinking green tea prevents mouth disease from chewing tobacco (denial of harm), (2) expressed skepticism toward scientific evidence that shisha contains harmful additives and that water filtration *does not* eliminate carcinogens (rejection of science), (3) suggested routine tasks like driving a car entail risks similar to pipe smoking (relative risk), or (4) asserted his status as a fitness expert while vaping (assertion of benefit). We selected videos that were popular without presenting expert sources. This selection allowed for the most stringent test of possible consequences of seemingly harmless amateur videos posted on YouTube. In addition to the lack of connection to a brand or sponsor, this selection also ensured a low probability of capturing commercial content.

**Figure 1:**
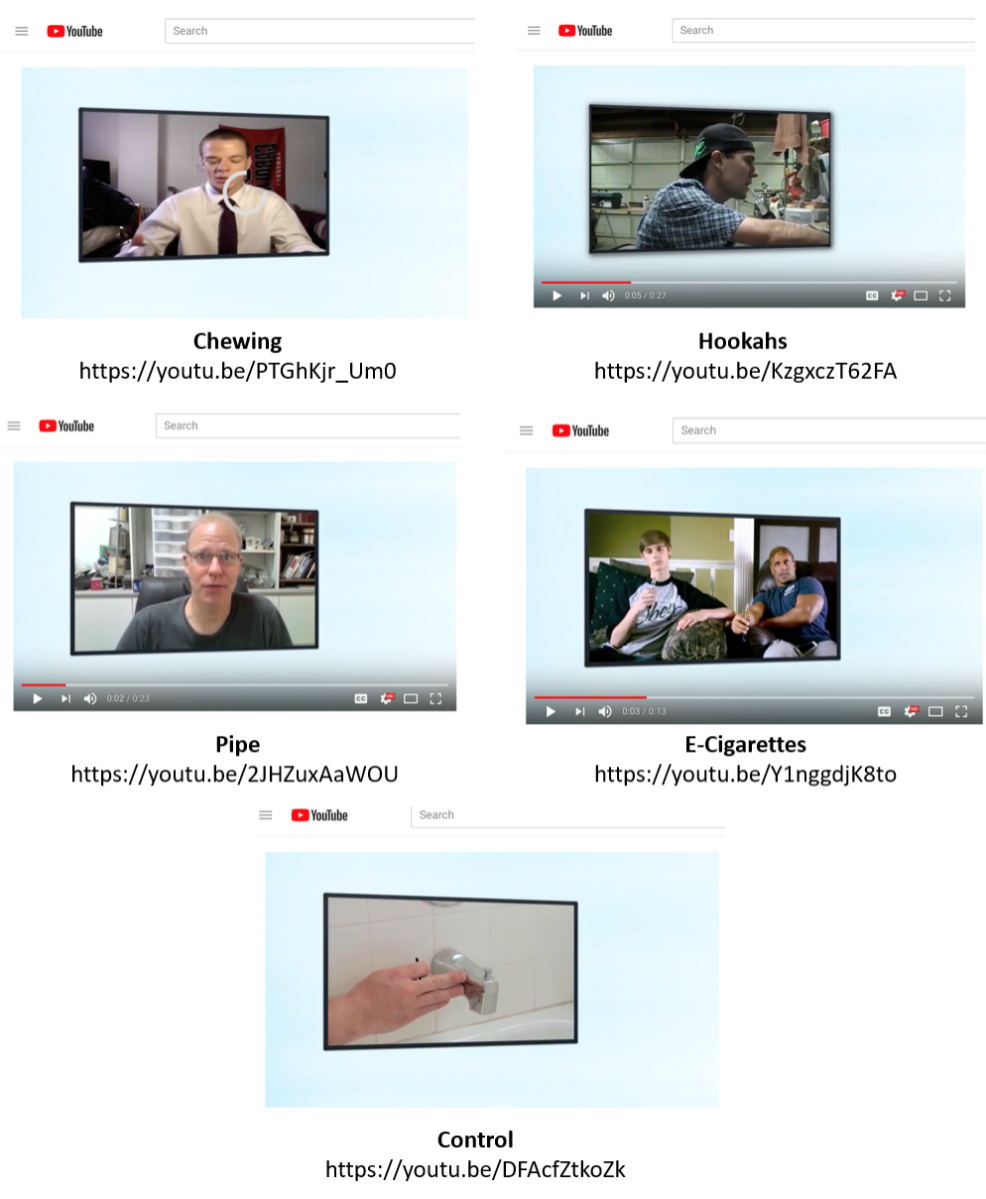
YouTube videos.

The control video was not related to health. This YouTube video featured a demonstration about replacing a shower faucet and was similar in duration and features to the experimental videos. Videos were cropped to minimize background and edited into brief (approximately 20 seconds) segments. Screenshots and links to the videos appear in Figure 1.

### Design

The conditions included five videos–four experimental videos and the control video– randomized between participants. Attitudes were measured after participants viewed one of the five videos. Specifically, in the experimental video conditions, we measured attitudes towards (1) chewing tobacco, (2) e-cigarettes, (3) hookahs, and (4) pipe smoking, depending on which was featured in the video. In the control video condition, we measured attitudes towards (1) chewing tobacco, (2) e-cigarettes, (3) hookahs, and (4) pipe smoking in all cases. Attitudes toward combustive cigarette smoking were measured in all conditions even though combustive cigarettes were not featured.

### Measures

#### Tobacco Use

We assessed use for the following products: cigarettes, cigars, pipe tobacco, chewing tobacco, hookah, and e-cigarettes. Participants first indicated which products they had ever tried. These measures were used to verify that the groups randomly assigned to conditions were indeed similar in experience.

#### Attitudes Toward Featured Tobacco Products

Attitudes toward tobacco use were measured with six semantic differential scales from 1 to 7, namely: *harmful* /*beneficial, wise/foolish, healthy/less healthy, enjoyable/unenjoyable, pleasant/unpleasant,* and *bad/good.* Participants in experimental conditions reported their attitudes toward the product mentioned in their video condition. Participants in control conditions reported their attitudes toward each of the five products mentioned in the experimental videos. Negative items were reverse scored. Attitude scales had good internal consistency (alpha>.70) and were averaged to form overall indexes of attitudes toward the featured product in experimental conditions and toward each product in control conditions. In addition to the overall attitude scale, we created scales for only positively worded items, and scales for only negatively worded reverse-scored items. The attitudes towards chewing tobacco, pipes, hookahs, and e-cigarettes in the control condition were averaged to compare with the attitudes in experimental conditions.

#### Attitudes Toward Combustive Cigarette Smoking

Combustive cigarettes were not the focal product in any of the videos but attitudes toward combustive cigarette smoking may still be indirectly promoted in videos featuring nontraditional forms of tobacco use. Thus, participants in experimental and control conditions were asked to report their attitudes toward smoking combustive cigarettes on scales from 1 to 7 anchored on the following adjectives: *harmful* /*beneficial, wise/foolish (R), healthy/less healthy (R), enjoyable/unenjoyable, pleasant/unpleasant (R),* and *bad/good*. The overall attitude scales after reverse-scoring the negative items had good internal consistency (alpha>.70) and were averaged to represent attitudes toward combustive cigarette smoking. In addition, we created a scale for only positively worded items, and another for only negatively worded reverse-scored items.

#### Credibility

Perceived credibility of the spokespersons with audio was measured with a 5-item scale with the following items: “To what extent do you agree that the person was speaking sincerely?”; “To what extent do you agree that the person is not worth listening to in the future?”; “To what extent do you agree that the person is a person who influenced my thinking on the matter?”; “To what extent do you agree that the person was communicating clearly?”; and “To what extent do you agree that the person is an expert on the topic?” These items had adequate internal consistency (alpha=.63) and were averaged as a measure of credibility.

## Results

### Sample Description and Comparability of Conditions

For descriptive purposes, we assessed use for the following products: cigarettes, pipe tobacco, chewing tobacco, hookahs, and e-cigarettes. We specifically asked participants, “Have you ever used any of the following tobacco products?”, after which they checked products that they had used. Table 1 contains the demographic and tobacco product usage description of the sample. The sample had considerable experience smoking combustive cigarettes, using hookahs, and using e-cigarettes but had little experience chewing tobacco and smoking pipes. As shown by the inferential statistics used to compare across experimental groups, there were no significant differences across the five video conditions in any of these characteristics. These analyses thus suggested that any experimental effects were due to the videos rather than a priori differences among groups of participants.

### Level of Credibility of the Spokesperson in Experimental Videos

The level of credibility of the spokesperson in the experimental videos was low to moderate, as judged by a mean credibility of 2.52 (SD 0.56), which differed significantly from the midpoint (3) of the scale, *t* (279)=–14.24, *P*<.001. The means and 95% confidence intervals were 2.30 (2.18-2.43) for chewing tobacco, 2.50 (2.38-2.62) for hookahs, 2.88 (2.76-3.01) for pipes, and 2.41 (2.29-2.53) for e-cigarettes; all suggestive of the low to moderate credibility of amateur sources.

### Message Effects on Attitudes

We estimated attitudes toward featured products across each specific experimental video and the control condition. As explained above, each experimental condition measured attitudes toward a different featured product, whereas the control condition measured attitudes towards all products, which were averaged for comparison. Table 2 presents these analyses; post hoc least significant difference (LSD) contrasts are represented with different subscripts. As shown, attitudes toward e-cigarettes and hookahs were more favorable following the experimental videos compared to the control video. In addition, the video about chewing tobacco produced more negative attitudes toward chewing tobacco that did the control video, and the video about pipe smoking did not differ from the control.

The effects on attitudes were investigated by conducting analyses of variance of attitudes toward the use of combustive cigarettes (never featured in the presented videos) as a function of experimental condition. Results from these analyses appear in the lower panel of Table 2 and show a significant effect of condition for both overall attitudes and positively worded attitude items. The omminbus effects on these attitude measures can be attributed to significant differences between the chewing and pipe videos, between the chewing and the e-cigarette videos, and between the hookah and the pipe videos. Although none of the videos differed significantly from the control videos, the pipe video produced the most favorable attitudes toward combustive cigarette smoking. Specifically, the pipe video led to more positive attitudes toward combustive smoking than did the chewing and the hookah videos, and the e-cigarette video led to more positive attitudes toward combustive cigarette smoking than did the chewing video.

**Table 1:** Descriptive statistics and comparisons across conditions*.*

Parameter		All	Chew	Hookah	Pipe	E-cigarettes	Control	Between-group statistic
Age (years), mean (SD)		21.98 (1.72)	22.15 (1.59)	22.04 (1.70)	22.02 (1.79)	21.80 (1.69)	21.87 (1.86)	0.47^a,b^
Male, n (%)		154 (44)	161 (46)	151 (43)	165 (47)	161 (46)	144 (41)	0.69^b,c^
**Race, n (%)**								19.37^b,d^
	White	256 (73)	207 (59)	273 (78)	259 (74)	280 (80)	224 (64)	
	Black	42 (12)	56 (16)	35 (10)	49 (14)	11 (3)	67 (19)	
	Native American	7 (2)	4 (1)	0 (0)	7 (2)	14 (4)	4 (1)	
	Asian	32 (9)	39 (11)	21 (6)	39 (11)	35 (10)	35 (10)	
	Other	14 (4)	11 (3)	25 (7)	7 (2)	11 (3)	21 (6)	
**Hispanic origin, n (%)**								2.87^b,c^
	Yes	42 (12)	35 (10)	14 (4)	28 (8)	32 (9)	39 (11)	
	No	308 (88)	315 (90)	336 (96)	322 (92)	319 (91)	312 (89)	
Ever chew, n (%)		42 (12)	46 (13)	35 (10)	60 (17)	39 (11)	35 (10)	2.08^b,c^
Ever hookah, n (%)		186 (53)	179 (51)	172 (49)	196 (56)	175 (50)	200 (57)	1.50^b,c^
Ever pipe, n (%)		42 (12)	39 (11)	42 (12)	39 (11)	49 (14)	46 (13)	0.52^b,c^
Ever e-cigarettes, n (%)		154 (44)	133 (38)	144 (41)	172 (49)	165 (47)	165 (47)	2.36^b,c^
Ever cigarettes, n (%)		196 (56)	179 (51)	182 (52)	217 (62)	210 (60)	200 (57)	2.76^b,c^

^a^ *F*_(4,345)._

^b^ns: not statistically significant.

^c^X^2^_(1,4)_.

^d^X^2^_(1,16)_.

**Table 2:** Means (95% CI) for attitudes as a function of video.

Attitudes		Video conditions					
		Chew	Hookah	Pipe	E-cigarette	Control	F_(4,315_)
**Attitudes towards products featured in videos**							
	Overall	1.53^a^ (1.27-1.80)	2.99^b^ (2.73-3.25)	2.49^c^ (2.21-2.76)	3.21^d^ (2.94-3.47)	2.57^b,c,e^ (2.21-2.93)	23.12^f^
	Positive items	1.46^a^ (1.18-1.74)	3.07^b^ (2.79-3.45)	2.40^c^ (2.11-2.70)	3.22^d^ (2.94-3.51)	2.57^b,c,e^ (2.19-2.95)	23.42^f^
	Negatively worded items (reversed-scored)	1.60^a^ 1.32-1.68)	2.92^b^ (2.65-3.19)	2.57^c^ (2.28-2.85)	3.19^d^ (2.92-3.47)	2.45^b,c,e^ (2.08-2.82)	18.51^f^
**Attitudes towards smoking**							
	Overall	1.89^a^ (1.67-2.12)	2.04^a,d^ (1.82-2.26)	2.39^b,d^ (2.16-2.62)	2.23^c,d^ (2.00-2.46)	2.13^a,d^ (1.91-2.36)	2.59^g^
	Positively worded items	1.85^a^ (1.55-2.06)	1.95^a,d^ (1.70-2.20)	2.33^b,d^ (2.07-2.60)	2.23^c,d^ (1.97-2.49)	2.06^a,d^ (1.80-2.32)	2.58^g^
	Negatively worded items (reversed-scored)	1.99^a^ (1.75-2.24)	2.13^a^ (1.89-2.38)	2.45^b^ (2.19-2.71)	2.24^a^ (1.99-2.49)	2.21^a^ (1.96-2.46)	1.70

^a-e^Within a row, different subscripts indicate statistically significant differences between cell means.

^f^*P*<.001.

^g^*P*<.05.

## Discussion

### Principal Findings

We examined the responses of young adults aged 18-24 years realted to four misleading portrayals of tobacco’s health consequences in popular YouTube videos. Results indicated that such material can increase positivity toward the featured products, such as e-cigarettes or hookah smoking (standardized difference Cohen *g* vs control video in each case was 0.38 and 0.37).

Although our study illustrates how potentially harmful content on social media may be studied, it has some limitations. We cannot generalize our findings to the many other videos that populate YouTube. We attempted to cover the major types of claims made in those videos, but there may well be others that are even more persuasive than the ones we identified. In addition, our findings with young adults may not generalize to adolescents, who may be even more susceptible to the claims made in these videos. Further research will be needed to assess this possibility.

This tendency for young tobacco consumers to respond credulously to misinformation on YouTube raises the possibility of tobacco use and exposure to misleading media exerting mutually reinforcing effects [31]. Finding tobacco-friendly material convincing and gratifying, recipients might seek out similar content, further bolstering self-justifying beliefs and prompting further selective exposure. In this regard, the aforementioned abundance of material on YouTube is cause for concern.

The present findings highlight the need to further study how new media sources such as YouTube affect tobacco knowledge, attitudes, and behavior. Although this study investigated misleading health portrayals only, content might also affect perceptions of tobacco products in other ways, both blatant and subtle, such as by modeling consumption [1-3], associating products with sex [16], or facilitating product acquisition [20]. Furthermore, the participatory, interactive, and self-selected nature of social media may enhance pro-tobacco media effects more readily and perniciously than was possible with traditional media.

### Policy Implications

The findings of YouTube video effects on positive attitudes toward hookahs and e-cigarettes should alert the public to the potential threats that these widely viewed videos can pose to youth and the health of the population. Attitudes toward hookahs and e-cigarettes can predict engagement in the behavior in the future [32-35], and essentially mimic the long-standing strategy of the tobacco industry to create favorable impressions of their products despite the harm they cause [36,37].

#### Difficulties Including User-Generated Postings Under the Total Ban on Tobacco Advertising, Promotion, and Sponsorship

The notion of regulating tobacco advertising is not new and stems from a large body of evidence on the powerful effects of the media on acceptance and use of tobacco. The Framework Convention on Tobacco Control organized by the World Health Organization has examined the media-effects evidence and recommended a total ban on tobacco advertising, promotion, and sponsorship [38]. The total ban is based on the principles listed in Textbox 1. A quick inspection of these principles highlights the difficulties that regulating Web 2.0 practices would pose. Web 2.0 is defined by an online environment in which users share information and build networks of users [39,40]. Thus, many of the user-generated social media postings are probably developed by independent citizens more likely to be motivated by the goal of achieving fame than by payments from the tobacco industry. In our study, the videos we selected had no associations with either a brand or a company and appeared to be amateur, and were judged to have limited credibility. Therefore, a direct connection between these developers and the tobacco industry is unlikely.

**Principles underlying a total ban on tobacco advertising, promotion, and sponsorship [53].**

1. It is well documented that tobacco advertising, promotion, and sponsorship increase tobacco use and that comprehensive bans on tobacco advertising, promotion, and sponsorship decrease tobacco use.
2. An effective ban on tobacco advertising, promotion, and sponsorship should, as recognized by Parties to the Convention in Articles 13.1 and 13.2, be comprehensive and applicable to all tobacco advertising, promotion, and sponsorship.
3. According to the definitions in Article 1 of the Convention, a comprehensive ban on all tobacco advertising, promotion, and sponsorship applies to all forms of commercial communication, recommendation, or action and all forms of contribution to any event, activity, or individual with the aim, effect, or likely effect of promoting a tobacco product or tobacco use either directly or indirectly.
4. A comprehensive ban on tobacco advertising, promotion, and sponsorship should include cross-border advertising, promotion, and sponsorship. This includes both outflowing advertising, promotion, and sponsorship (originating from a Party’s territory) and in-flowing advertising, promotion, and sponsorship (entering a Party’s territory).
5. To be effective, a comprehensive ban should address all persons or entities involved in the production, placement, and/or dissemination of tobacco advertising, promotion, and sponsorship.
6. Effective monitoring, enforcement, and sanctions supported and facilitated by strong public education and community awareness programs are essential for implementation of a comprehensive ban on tobacco advertising, promotion, and sponsorship.
7. Civil society has a central role in building support for, developing, and ensuring compliance with laws addressing tobacco advertising, promotion, and sponsorship, and it should be included as an active partner in this process.
8. Effective international cooperation is fundamental to the elimination of both domestic and cross-border tobacco advertising, promotion, and sponsorship.

#### Obstacles to Banning Internet Contents in the United States

Most countries impose some regulations on internet contents following the principle of protecting children from potentially harmful contents [41]. However, in the United States, restricting online interactions is perceived as violating freedom of speech and as, “throwing the baby with the bath water.” That is, restricting freedom of speech in any way may open the door to censorship, and in fact some of the countries that control internet contents are motivated by political reasons [42]. The Fifth Amendment would also be violated by imposing a total ban on internet tobacco contents. The Fifth Amendment protects the right to act in ways that are not self-incriminating and thus make all internet use private.

#### Voluntary Ban by YouTube

A more promising measure to reduce postings that promote tobacco use is for social media platforms to restrict postings. Verifying that amateur videos can still have measurable impacts on attitudes toward tobacco use should be a sufficient demonstration that responsible businesses must advocate for socially beneficial behavior in their premises. For example, in 2015, Facebook expanded the list of contents that the company is allowed to remove, which includes violent materials and postings that are degrading to specific social groups (eg, women) [43]. Following in Facebook's steps, in 2017 YouTube updated the guidelines of acceptable contents as follows:

Chiefly, the video site will not show advertising of “hateful” content that “promotes discrimination or disparages or humiliates an individual or group of people,” it said. Also barred from running ads are videos that involve “family entertainment characters” engaging in inappropriate behavior, and those that carry messages that demean or are incendiary.

44

Moving from these guidelines to banning advertising of such products with no known benefits (eg, tobacco) would be easy and beneficial to society.

#### User Norms and Boycotting Sites

User boycott of sites that allow tobacco promotion would be another reasonable step. In 2016, several proposals were suggested to boycott Facebook following the company's involvement in dissemination of fake news during the American presidential campaign [45]. These proposals were likely influenced by reports that 67% of Americans believed that Facebook should have done more to prevent the spread of fake news during the 2016 US presidential election on their site [46]. These public responses prompted the company's decision to limit fake news, although the success of these measures remains to be seen [47]. Even more relevant to our analysis, during the first half of 2017, YouTube faced a boycott from advertisers who refused to have their ads displayed next to the many hate speech videos that populate the site [48]. The boycott was effective at influencing their 2017 posting policies, which we covered above.

#### Electronic Filtering or Control Methods

Content-limited (or filtered) internet service providers allow subscribers to opt into specific websites or set mandatory restrictions for all subscribers. These are the most extreme forms of filtering and enable government, organizational, or parental control over the contents viewed by subscribers [49]. Less dramatic methods involve network-based filtering, in which software is installed to control content within a network such as a home or school [50]. Filtering can also be done by domain, by user, or by a combination of user and domain, all of which may be implemented for tobacco-related materials [50].

#### Debunking and Recipient Training Approaches

Future research should also explore ways to debunk the misleading claims found in YouTube videos to render these materials ineffective. A recent meta-analysis of debunking messages [51,52] suggested that detailed forms of debunking, as well as an active audience, are key to effective corrections [53]. Furthermore, resistance training has been identified as key to reducing susceptibility to peer influences on smoking [54]. In the context of our findings, young adults should be trained in identifying and resisting contents that appear on social media, but share many of the characteristics of peer pressure to use unhealthy products.

## Abbreviations

e-cigarette: electronic cigarette
LSD: least significant difference
ns: not statistically significant
